# Yeast-like chronological senescence in mammalian cells: phenomenon, mechanism and pharmacological suppression

**DOI:** 10.18632/aging.100402

**Published:** 2011-12-10

**Authors:** Olga V. Leontieva, Mikhail V. Blagosklonny

**Affiliations:** ^1^ Department of Cell Stress Biology, Roswell Park Cancer Institute, BLSC, L3-312, Buffalo, NY, 14263, USA

**Keywords:** chronological aging, senescence, metabolism, rapamycin, mTOR, lactate

## Abstract

In yeast, chronological senescence (CS) is defined as loss of viability in stationary culture. Although its relevance to the organismal aging remained unclear, yeast CS was one of the most fruitful models in aging research. Here we described a mammalian replica of yeast CS: loss of viability of overgrown “yellow” cancer cell culture. In a density and time (chronological)-dependent manner, cell culture loses the ability to re-grow in fresh medium. Rapamycin dramatically decelerated CS. Loss of viability was caused by acidification of the medium by lactic acid (lactate). Rapamycin decreased production of lactate, making conditioned medium (CM) less deadly. Both deadly CM and lactate caused loss of viability in low cell density, not preventable by either rapamycin or additional glucose. Also, NAC, LY294002, U0126, GSK733, which all indirectly inhibit mTOR and have been shown to suppress the senescent phenotype in traditional models of mammalian cell senescence, also decreased lactate production and decelerated CS. We discuss that although CS does not mimic organismal aging, the same signal transduction pathways that drive CS also drive aging.

## INTRODUCTION

In yeast, chronological senescence (CS) is defined as loss of viability of yeast cells grown in confluent state [1-10]. Viability is determined as the ability to resume replication in fresh medium. CS is genetically regulated and inactivation of numerous genes including TOR (Target of Rapamycin) extends lifespan [2, 11-23]. Furthermore, rapamycin, an inhibitor of TOR, decelerates CS in yeast [24]. Noteworthy, the TOR (target of rapamycin) pathway is involved in aging of variety of species from worm to mammals [25-28]. Some other genes identified in the yeast model turned out to be involved in organismal aging and age-related diseases in mammals [29-38]. CS is often compared to aging of postmitotic cells in the organism. However, as recently discussed, the link between CS and organismal aging is not immediately apparent but indirectly relies on common genetic pathways [39].

Since yeasts are unicellular organisms, CS should be compared with some form of cellular senescence in cell culture. There are two types of senescence of mammalian cells in culture: replicative and accelerated (also known as premature or stress-induced senescence). Yeast replicative senescence corresponds to replicative senescence in rodent cells. Then at first glance, yeast CS is analogous to accelerated cellular senescence. The analogy is seemingly strengthened by the involvement of mTOR in senescent phenotype of mammalian cells [40-46]. In proliferating cells, mTOR is active, thus driving cell growth in size, which is balanced by cell division. When cell cycle is blocked by p21 or p16, for instance, but mTOR is still active, then mTOR drives cellular senescence [40, 46, 47]. By deactivating mTOR, rapamycin prevents conversion of quiescence into senescence or, in other words, prevents gerogenic conversion (geroconversion) during cell cycle arrest [41, 46].

Yet accelerated senescence is not analogous to yeast CS. Accelerated cellular senescence is not caused by medium acidification. Senescent cells are large, flat, highly viable and apoptosis-resistant**.**Furthermore, gero- conversion occurs in low cell densities, whereas in high density mTOR is spontaneously deactivated thus suppressing geroconversion (manuscript in preparation). So there is no known mammalian analogy to yeast CS. Given that yeast aging research has been so fruitful in identification of pathways involved in organismal aging, [1, 3, 48-51] it is important to recognize relevant mammalian cell model for CS.

We realized that replica of CS is so trivial that it remains unnamed and ignored, although this phenomenon is known to any cancer researcher. If a flask with cancer cells is forgotten (neglected) in the incubator, cells become over-confluent, overgrow, and loose viability. And this is exactly what would be called chronological aging (in yeast). Here we investigated mechanisms of chronological senescence (**CS**) in human cells and its pharmacological suppression.

## RESULTS

### TOR-dependent CS in HT-p21-9 cells

**Figure 1:**
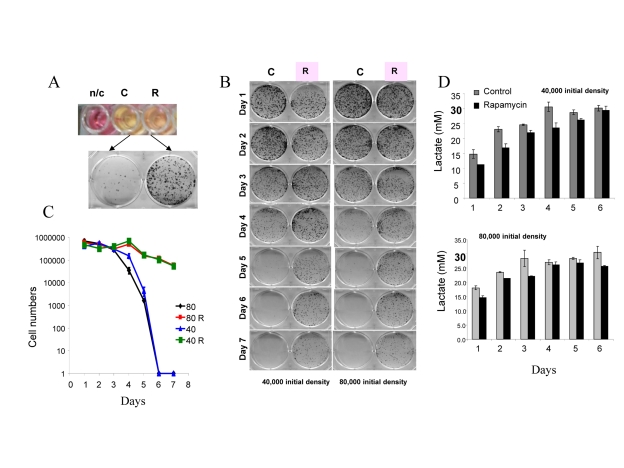
Defining mTOR-dependent CS (**A**) Measurement of CS. 80000 HT-p21-9 cells were plated in 0.2 ml medium per well in 96-well plate with 500 nM rapamycin (R) or without rapamycin – control (C). After 4 days, the plate was photographed to record color of media (i.e. pH). The color of medium without cells is shown for comparison n/c (no cells). Then cells from each well were split into larger wells of 6-well plates. Detailed description: medium (together with floating cells) was aspirated and adherent cells were trypsinized in 0.2 ml of trypsin. Equal volume (a 4 μl aliquot of cell culture) or 2% of total adherent (live) cells was plated in 4 ml of fresh medium in 6-well plates. After 7 days, colonies were stained and photographed. A number of colonies is a measure of viability of the stationary culture. (**B**) Time-dependent (chronological) loss of replicative viability. Cells were plated at initial density of 40000 and 80000 cells per well in 96-well plates without (C) or with rapamycin (R) as shown in panel A. After indicated time, cells were trypzinised and equal volume of adherent cells (2%) were re-plated in 6 well plates and allowed to form colonies for 7 days. (**C**) Cells from plates shown in panel B were trypsinized and counted to precisely quantify replicative viability. (**D**) Levels of lactate in conditioned medium. Cells were plated at initial density of 40000 and 80000 cells per well in 96-well plates without (C) or with rapamycin (R) as shown in panel A. After indicated time, concentration of lactate was measured in conditioned media.

For initial experiments we chose HT-p21-9 cells, because these cells were extensively studied as a model of conventional cellular senescence [52, 41]. Importantly, these cells rapidly turn media yellow. Acidification of the medium is evident by transition of the color of phenol red (a pH indicator) from pink-red to yellow over the pH range 7.4 to 6.8. The color of phenol red is a convenient measure of pH. In dense cell culture, the medium becomes acidic (yellow) (Fig. 1A: top panel – control (C). Rapamycin renders the color pinkish. After recording the color by photography, media were collected for further analysis (lactate levels, cytotoxicity) and equal volumes of trypsinized cells were re-plated in larger plates in fresh medium (Fig. 1 A, bottom panel) and colonies were allowed to grow for 7 days. In control, cells lost the replicative capacity and did not form colonies in fresh medium (Fig. 1A, bottom panel). Rapamycin prevented loss of viability (Fig. 1A). Loss of viability, over time (Fig. 1B), is chronological senescence (CS). At initial cell densities of 40,000 and 80,000 cells per well (Fig. 1 B), the major drop in viability occurred at day 5 and day 4, respectively (Fig. 1B). The most dramatic effect of rapamycin was observed, when control cultures almost completely lost their viability. To quantify the viability, we counted cells after 7 days of re-growth in fresh culture (Fig. 1C). There was a dramatic decrease in viability after 4 and 5 days in control wells, which was prevented by rapamycin (Fig. 1 C).

### Time course of lactate production

At both cell densities, lactic acid (LA) concentrations reached the maximal level of ~30 mM on day 3 and day 4, respectively (Fig. 1D). Noteworthy, these maximal levels of LA were achieved a day before the major loss of viability (Fig. 1 D versus C). Rapamycin decreased levels of LA, so that LA concentration approximated to the maximal level by day 6-7. A comparison of LA levels and CS time points suggests that levels of LA above 30 mM may make medium toxic: then cells loose viability and this prevents further accumulation of LA.

### Protection by rapamycin is indirect

**Figure 2:**
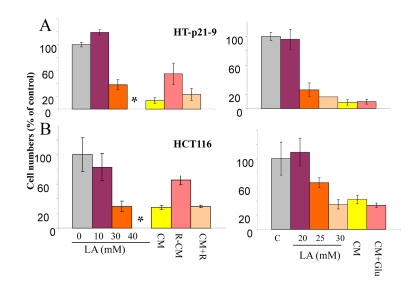
Cytotoxicity of conditioned medium (CM) and lactate HT-p21-9 cells (A) and HCT116 cells (B) were plated in 96-well plates at low density (2000 cells/well) and treated with a range of lactic acid (LA) concentrations or conditioned medium (CM), collected from overgrown HT-p21-9 cell culture. After 3 days, medium was changed to fresh medium and cells were allowed to proliferate for 5 days and then number of viable cells was determined using MTT assay. CM, R-CM, and CM+R were prepared as described in Methods.

Next, we tested the effect of conditioned medium (CM) that was collected from the dense culture and applied to cells plated at low density. Such CM was deadly, causing loss of viability in low cell density. This allowed us to investigate the mechanism of action of rapamycin: direct versus indirect. One possibility is that rapamycin protects cells directly by increasing resistance to cell death caused by deadly CM. Another possibility is that CM produced in the presence of rapamycin would be less deadly. We tested CM produced in the presence of rapamycin (R-CM) versus CM with rapamycin being added after CM collection (CM+R). Both types of CM were added to low density HT-p21-9 and HCT116 cells for 3 days and then the cells were grown in fresh medium. CM+R was as toxic as CM, whereas R-CM was significantly less toxic (Fig. 2). We conclude that rapamycin does not protect cells directly, but instead changes the property of CM. As we have already shown, rapamycin decreased levels of LA (Fig. 1D).

### Lactate poisoning

Next we tested whether LA is toxic. Addition of 40 mM lactate to fresh medium rendered it “absolutely deadly” (Fig. 2 A). Viability IC_50_ was around 20-30 mM LA, depending on cell line. Noteworthy, color of the medium containing 30 mM lactate was similar to yellow color of CM in the stationary culture, when cells undergo CS. This was confirmed by measurements of LA in such deadly CM (34.6 ± 1 mM). So LA levels slightly above 30 mM were maximally achievable, killing cells and thus preventing further accumulation of LA.

There are 2 possibilities that are not mutually exclusive: 1) **LA** is toxic by itself and is the only cause of CS. Rapamycin, which decreases levels of LA, simultaneously decreases toxicity of CM; 2) production of 30 mM lactate requires metabolism of 15 mM glucose. In principle, exhaustion of glucose may contribute to CM toxicity. However, addition of glucose to CM did not decrease its toxicity (Fig. 2 B).

Thus, LA was the main cause of toxicity. Next, we tested whether this toxicity was due to LA acidity. To neutralize pH, we added NaOH to cells plated in high density after one day of cultivation. At concentrations (0.5-1 mM) NaOH shifted the medium color from yellow to pink and decreased CS (Fig. S1). We conclude that it is acidity of LA that is deleterious for the cells.

### Gerosuppressants decrease lactate levels and CS

**Figure 3:**
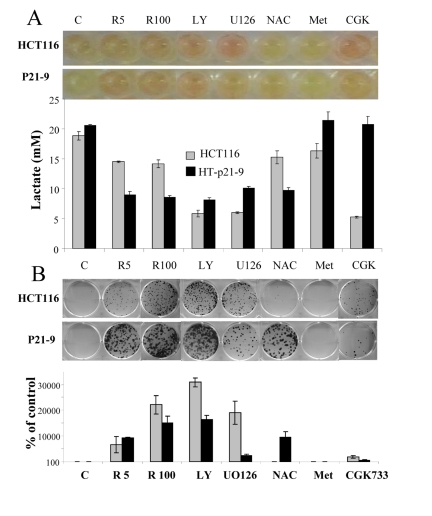
Testing potential gero-suppressants HCT116 cells and HT-p21-9 cells were plated at 80000 per well in 96 well plates with indicated agents: 5 nM and 100 nM rapamycin (R5 and R 100), 10 μM LY294002 (LY), UO126, and CGK733, 2 mM NAC. (**A**) After 4 days, the plates were photographed (upper panel) and lactate concentrations were measured in media (lower panel). (**B**) An equal volume of attached cells (an aliquot of 2%) was plated in 6 well plates. After 7 days, colonies were stained with Crystal Violet (upper panel) or total numbers of cells per well were determined by counting (low panel).

Recently we have demonstrated that agents that deactivate the mTOR pathway decelerate premature (p21-induced) senescence in HT-p21-9 cells [41, 53, 54]. Given that these agents suppress the conversion from quiescence to senescence (geroconversion), we named them gero-suppressants [55]. Here we evaluated the effect of gero-suppressants on CS, using rapamycin as a positive control (Fig. 3). Inhibitors of PI-3K and MEK, LY294002 and U0126, respectively, decreased acidosis (Fig. 3 A), lactic acid production (Fig. 3 B) and dramatically decreased CS in HT-p21-9 and HCT116 cells (Fig. 3 C-D). Suppression of CS correlated with inhibition of LA production in both cell lines. The antioxidant N-acetyl-L-cysteine (NAC) decreased LA production preferentially in HT-p21-9 cells and also prevented CS in the same cell line. CGK733, in contrast, was preferentially effective in HCT116 cells (Fig. 3). Finally, metformin did not affect either LA levels or CS in any cell lines (Fig. 3).

**Figure 4:**
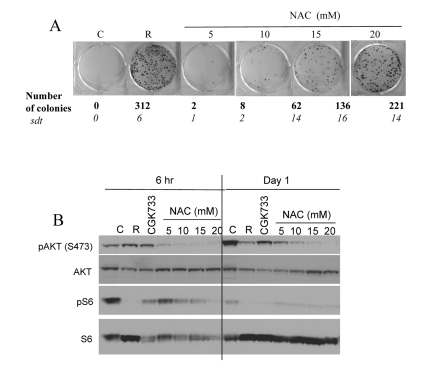
Effects of NAC and CGK733 on CS and mTOR (**A**) HT-p21-9 cells were plated at 40000 per well in 96 well plates with indicated agents: 500 nM rapamycin (R), 5-20 mM NAC and incubated for 6 days. On Day 6 replicative viability of cells was measured by colony formation (8 days) as described in legend for Figure 1. (**B**) HT-p21-9 cells were plated at high density with indicated agents or left untreated (C) and allowed to attach for 6 hrs. Then, one set was lysed (Day 0, 6 hr) and second set was lysed after 24 hrs treatment (Day 1). Immunoblot was performed using indicated antibodies.

Noteworthy, free radicals and oxidative stress can activate Akt/mTOR and vice versa active TOR may promote ROS [9, 56]. Therefore, inhibition of ROS by NAC is expected to decrease not only ROS levels but also mTOR activity. In fact, NAC inhibited CS in a dose-dependent manner with maximal effect at 20 mM (higher concentrations were toxic) (Fig. 4 A). In high cell density, mTOR was spontaneously deactivated after first day in culture. NAC inhibited the Akt/mTOR pathway by 6 hrs (Fig. 4B, 6 hrs), before mTOR becomes spontaneously deactivated. Noteworthy, the ATM inhibitor CGK733 also inhibited mTOR (Fig. 4B).

At high cell density, mTOR is shut down after 1 day in culture (Fig. 4 and Fig. S3). Still, rapamycin and other mTOR inhibitors suppressed CS very effectively. This suggests that the activity of mTOR during the first day (before spontaneous deactivation of mTOR) determines cell fate.

### Effects of pre-conditions

We have noticed that time course of CS was slightly variable in different experiments (compare HCT116 cells in Fig. 3 and Fig. 6), even if cells were plated at the same densities and conditions. We reasoned that this may be due to different pre-conditions, given that mTOR becomes spontaneously deactivated in culture over time. This suggests that cells from the fresh culture will lose viability faster compared with those from the old culture. We tested this prediction. 80,000 cells from either old or fresh culture were plated in 96-wells in fresh medium and cultivated for the same time before measuring their viability. In fact, cells derived from “old pre-culture” retained viability longer (Fig. S2). Whereas rapamycin prevented CS in cells derived from fresh pre-culture, its effect was minimal in old pre-culture cells (Fig. S2). This result is in agreement with the data that mTOR is already inhibited in old pre-culture.

### Lactate production *vs* apoptosis-reluctance as determinant of CS

**Figure 5:**
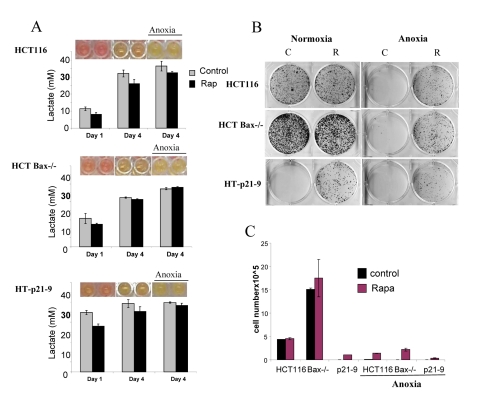
Effects of glycolytic phenotype, apoptosis resistance and anoxia on CS in very high initial cell density HCT116, HCT-Bax−/− cells and HT-p21-9 cells) were plated at 80000 per well in 96 well plates and next day 1 set of plates was placed under anoxia and 2 nd set was incubated in normoxia. If indicated, cells were treated with 100 nM rapamycin (R). (**A**) Wells were photographed (upper panels) and LA (lower panels) was measured after 1 day (D 1) or on the last 4^th^ day (D 4) of culture. (**B**) Replicative viability. On day 4, equal aliquots of attached cells were plated in 6 well plates. After 7 days, colonies were stained with Crystal Violet (upper panel) or total numbers of cells per well were determined by counting (lower panel).

**Figure 6:**
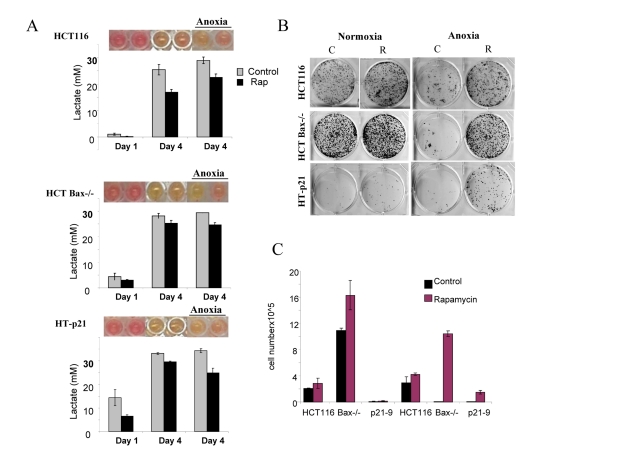
Effects of glycolytic phenotype, apoptosis resistance and anoxia on CS in medium initial cell density HCT116, HCT Bax−/− cells and HT-p21-9 cells were plated at 20000 per well in 96 well plates and after 1 day were placed in normoxia or anoxia. If indicated, cells were treated with 100 nM rapamycin (R). (**A**) Wells were photographed (upper panels) and LA (lower panels) was measured on 1 day (D 1) and on the last 4^th^ day (D 4) of culture. (**B**) Replicative viability. On day 8, equal aliquots of attached cells were plated in 6 well plates. After 7 days, colonies were stained with Crystal Violet (upper panel) or total numbers of cells per well were determined by counting (lower panel).

We next compared 3 cell lines: HT-p21-9, HCT116 and HCT116-Bax−/−, a clone of HCT116 cells lacking Bax (Fig. 5 and 6). HCT-Bax−/− cells are apoptosis-reluctant [57, 58]. Cells were plated in 2 cell densities (80,000 and 20,000 cells per well). At high cell density on day 4, HT-p21-9 cells lost viability, which was prevented by rapamycin (Fig. 5B). In contrast, HCT116 and HCT-Bax−/− cells retained viability at that time point (in this particular experiment).

**Figure 7:**
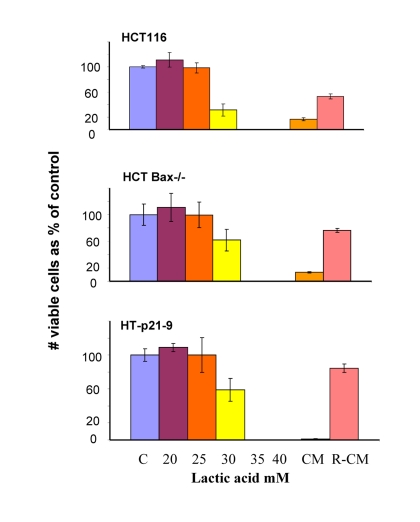
Lactate resistance of HT-p21-9, HCT116 and HCT116-Bax−/− cells HCT116 cells, HCT-Bax−/− and HT-p21-9 cells were plated in 96-well plates at low density (2000 cells/well) and treated with a range of lactic acid (LA) concentrations or conditioned medium (CM). After 3 days, medium was changed to fresh medium and cells were allowed to proliferate for 5 days and then numbers of live cells were determined using MTT assay. CM and R-CM were prepared as described in Methods.

In comparison with HCT116 cells, HT-p21-9 cells produced considerably more LA during the first day, which reached sub-lethal levels at that time (Fig. 5A). This demonstrated that levels of LA reached on the first day determine CS. However, HCT-Bax−/− cells, which were less prone to CS produced more lactate than HCT116 parental cells, indicating that resistance to apoptosis can also determine the viability. That was confirmed by direct testing of lactate on cell viability: HCT-Bax−/− cells appeared to be slightly more resistant to 30 mM LA than parental cells (Fig. 7). Still, the difference in resistance was relatively small. The major factor that determined CS was the rate of LA production during the first day in culture (Fig. 5, 6). At low cell density, the difference in LA production was the most prominent (Fig. 6A). HT-p21-9 cells were the most glycolytic, whereas HCT116 cells were the least glycolytic (Fig. 6A, day 1). By day 4, HT-p21-9 cells produced near-lethal levels of lactate (Fig. 6A). Accordingly, HT-p21-9 cells lost viability by day 8 (Fig. 6 B, C).

### A forced increase in lactate levels accelerated CS

If mTOR-dependent lactate production (rather than the activity of mTOR per se) is responsible for CS, then anoxia will accelerate CS. Anoxia and hypoxia induce (hypoxia-inducible factor) HIF-1 and this effect is not blocked by rapamycin (Fig. S3). Furthermore, hypoxia/anoxia decreases the activity of the mTOR pathway [59, 60]. So in anoxia LA production is forced by HIF-1. On the other hand, hypoxia/anoxia may diminish mTOR-dependent glycolysis by deactivating mTOR. Not surprisingly, relationship between aging and hypoxia are complex [61].

In anoxia experiments, cells were cultivated without oxygen for 3 days: from day 1 to day 4 (Fig. 5, 6). At high cell density on day 4, HT-p21-9 cells lost viability, which partially was prevented by rapamycin (Fig. 5 B, C). Furthermore, even HCT116 and HCT-Bax−/− cells lost viability in anoxia but not in normoxia (Fig. 5, 6). Yet, final levels of LA were almost identical in both normoxia and anoxia because CS prevented further accumulation of lactate (Fig. 5A, Fig 6A).

Rapamycin decelerated CS under both normoxia and anoxia. This might be due to a decrease in lactate production on the first day (especially in high density cultures, Fig. 5A) before cells were placed in anoxia. Alternatively, rapamycin may decrease cellular metabolism in anoxia, which would indicate that the effect of rapamycin was not due to its effects on respiration. Experiments here were not intended to determine whether the protective effect of rapamycin was independent of respiration. Further studies are under way to determine the effect of rapamycin on glycolysis under anoxia versus normoxia.

### Suppression of CS by rapamycin was not due to its cytostatic effect

**Figure 8:**
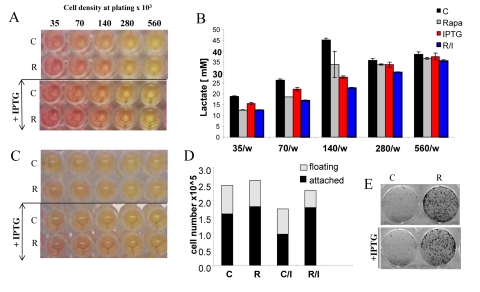
Effects of IPTG, rapamycin and initial cell densities on CS HT-p21-9 cells were plated at indicated densities (x10^3^) in 96-well plates in the presence of IPTG and/or 500 nM rapamycin (R) or left untreated (C). (**A**) On day 1, plates were photographed. (**B**) On Day 1 lactate concentrations were measured in media. (**C**) On day 4 plates were photographed. (**D**) On Day 4 alive (attached) and dead (floating) cells were counted in wells with initial plating density of 140,000 cells/well. (**E**) Replicative viability of attached cells was measured as described in Materials and Methods and legend for Figure 1.

Rapamycin is moderately cytostatic in some cell lines including HT-p21-9 cells. However, the cytostatic effect in low cell density cannot account for deceleration of CS in high cell density. First, at high cell densities with a limited proliferation window, the cytostatic effect of rapamycin was undetectable. We plated HT-p21-9 cells in a wide range of initial cell densities (Fig. 8) from 35000 to 560000 cells per well with and without IPTG. In HT-p21-9 cells, IPTG induces ectopic p21 and causes cell cycle arrest [52], thus ensuring a constant number of cells.

**Figure 9:**
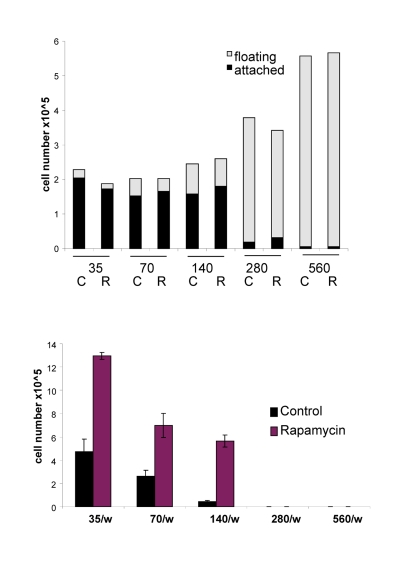
Effects of initial density and rapamycin on cell proliferation, survival and replicative viability Experiment was performed as described in the legend for Figure 8 (Upper panel). Cell survival. Alive (attached) and dead (floating) cells were counted in all wells on Day 4. (Lower panel). Replicative viability. In all samples, attached cells were replated in the fresh medium in low density and viability was measured by trypsinizing colonies and counting cells.

The acidity (yellow color) of the medium was proportional to initial cell numbers up to 140000 (Fig. 8 A) and corresponded to deadly levels of LA (Fig. 8 B). Rapamycin decreased levels of lactate. Noteworthy, highest levels of lactate were achieved at initial density 140000 (Fig. 8 B), but not at higher densities. At initial density of 280000 and 560000, all cells died within several days (Fig. 9, upper panel). These cells were floating and did not produce any colonies (Fig. 9, lower panel). On day 4, numbers of adherent (alive) cells were approximately identical at initial plating densities between 35000 and 140000 (Fig. 9, upper panel). This means that cells underwent 2, 1, and 0 divisions when plated at initial densities of 35000, 70000 and 140000, respectively. Most importantly, rapamycin did not affect the number of live cells (Fig. 9, upper panel); even though it decreased LA levels (Fig. 8 B). Despite rapamycin did not have an effect on the number of live cells, it dramatically increased the number of cells with replicative potential (Fig. 9, lower panel).

**Figure 10:**
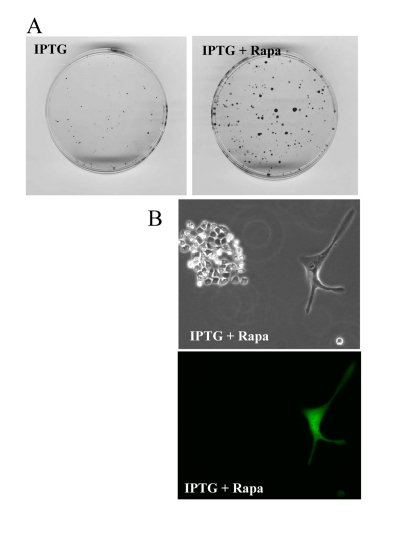
Rapamycin prevents CS of a few indicator cells by indirect effects on abundant cells A mixture of 2000 HCT116 cells (indicator, non-green cells) and 80000 HT-p21-9 (GFP-expressing, green cells) were plated in 96-well plate with 50 μg/ml IPTG with or without 500 nM rapamycin. After 4 days, equal aliquots of attached cells were re-plated in 60 mm dishes in fresh medium with IPTG to prevent proliferation of HT-p21-9 cells and to allow only viable indicator cells to form colonies. (**A**) After 7 days, colonies were stained. (**B**) Microphotograph of live cells before staining. A colony of HCT116 cells with a solitary large HT-p21-9 cell (labeled with GFP) is shown.

At initial density of 140000 cells, cells did not proliferate and did not die (Fig. 9). At this density, the level of lactate was maximal as well as the effect of rapamycin on CS (Fig. 8). Furthermore, we excluded the cytostatic effect of rapamycin by mixing IPTG- arrested HT-p21-9 (GFP-positive) cells with a few HCT116 cells, termed here as indicator cells. Since HT-p21-9 cells were already arrested by IPTG, rapamycin could not possibly arrest them further. The indicator HCT116 cells are not sensitive to cytostatic effects of rapamycin. Even if rapamycin could decrease proliferation of the indicator HCT116 cells, the small number of indicator cells could not significantly contribute to lactate production. We treated the cell mixture (80000 HT-p21-9 cells mixed with 2000 indicator cells) with IPTG (Fig. 10). After 4 days of incubation, small aliquots of cells were re-plated in fresh medium with IPTG to preclude proliferation of HT-p21-9 cells. As shown in Figure 10A, rapamycin dramatically increased a number of colonies. It was confirmed by microscopy that only HCT116 cells formed colonies, whereas IPTG-arrested HT-p21-9 (green) cells became senescent (large morphology of green cells) (Fig. 10B).

## DISCUSSION

Here we described a mammalian model of yeast-like chronological senescence (CS). Regardless of how trivial this phenomenon is, it is the replica of yeast CS.

For both yeast and cancer cells, CS can be defined as loss of replicative viability in a stationary culture.

Cancer cells with high glycolysis resemble yeast cells [62-65]. The most predictive sign of CS is yellow color of the medium, which indicates pH. Lactate and conditioned medium (CM) by were deadly in cells plated in low density. Furthermore, levels of lactate that caused CS coincided with maximally reachable lactate levels in the culture. Neutralization of the acidity prevented CS. Similarly, in some studies in yeast, loss of replicative ability was attributed to acidosis [39, 66-68]. The only difference is that mammalian cells produce lactic acid instead of acetic acid. Rapamycin decelerated CS. Rapamycin decreased lactate accumulation, keeping lactate levels below deadly levels. In other words, CS is a metabolic self-destruction or hypermetabolism-induced loss of replicative ability. By producing lactate, cells poison themselves and cannot resume replication in the fresh medium.

**Figure 11:**
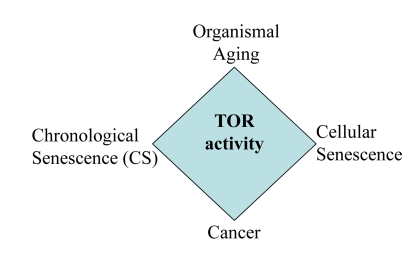
The relationship between CS and other TOR-dependent phenomena Common signaling pathways such as TOR are involved in cancer, aging, cellular senescence and CS.

Still mechanisms of yeast CS are widely disputed and may depend on culture conditions [9, 18, 19, 22, 66-68]. Perhaps, mechanisms of mammalian CS may vary depending on initial cell densities, cell line, the propensity to apoptosis, levels of nutrients in the medium, initial mTOR activity, levels of oxygen and culture conditions prior to experiment. Also, we emphasize that our study was not intended to address the mechanisms of yeast CS. It neither resolves nor complicates the current debate in yeast aging research. The main goal of this study was to characterize “yeast-like” senescence in human cells and its pharmacological manipulation and also to address the question whether CS mimics cellular aging in the organism. CS mimics tumor necrosis rather than aging of post-mitotic cells. So, literally, CS is not a physiological model of organismal aging. But the same signaling pathways (such as TOR) are involved in CS and aging (Fig. 11), making the model useful for drug and gene discovery.

We have shown that agents that decrease lactate production decelerate CS. Specifically, agents that directly or indirectly inhibit mTOR such as rapamycin, LY294002, U106 and to a lesser extend NAC, all suppressed CS. What all these agents have in common is that they inhibit the mTOR pathway. Thus, the same agents that suppress CS also were shown to suppress the senescent phenotype or conversion from quiescence to senescence during cell cycle arrest in traditional models of cellular senescence [46, 53].

Activation of growth factor receptors, Ras, Raf, PI3K, Akt, which all activate mTOR, are most common alterations in cancer [69-83]. These growth-promoting pathways also drive senescent phenotype, when the cell cycle is blocked [40, 53, 55]. The same pathways confirm highly glycolytic phenotype [84, 85], rendering cells prone to lactate-induced CS. And the same oncogenic pathways are involved in aging from yeast to mammals [11, 86-88]. And tumor suppressors (including p53) that inhibit the mTOR pathway are aging-suppressors [44, 89-93].

In line with previous proposals [11, 39], our work suggests that the same signaling pathways that are known to drive aging are also involved in glycolytic phenotype, which determines CS. Therefore, genetic and pharmacological manipulations that decelerate CS may also affect physiological aging. The same pathways (Ras, MEK and PI-3 K/Akt/mTOR) that render cells malignant and glycolytic are involved in aging. So the same agents that inhibit CS can also suppress malignant metabolism and organismal aging. In addition such agents may be beneficial during acute ischemia (as well as re-oxygenation) to prevent necrosis. Lactic acidosis is one of the complications of diabetes. Also, overproduction of lactate by tumors can lead to fatal lactic acidosis in cancer patients [94-96]. We suggest that rapamycin may be used to treat lactic acidosis both in diabetes and in cancer patients. Interestingly, stromal cells also overproduced lactate, thereby feeding the tumor [97-99]. We suggest that systemic rapamycin may decrease lactate production in the stroma.

Pro-aging pathways are conserved in evolution. This may explain why screening for agents that decelerate yeast CS could be useful against age-related diseases, aging and cancer. In comparison with yeast, human cells are more relevant to human aging and drug discovery.

In conclusion, CS is relevant to aging not because CS happens during physiological aging in the organism, but because the common genes and signaling pathways determine both CS and aging (Fig. 11). Here we discussed CS from the point of view of the aging research, as an analogy to yeast CS. From the cancer research point of view, we will discuss the implication of CS for tumor progression, aggressiveness and cancer therapy (Leontieva et al, Oncotarget, in press).

## MATERIALS AND METHODS

### Cell lines and reagents

HT-p21-9 cells, derived from HT1080 human fibrosarcoma cells (ATCC, Manassas, VA), provided by Igor Roninson, were previously described [52, 41]. HT1080-p21-9 cells were cultured in high-glucose DMEM without pyruvate supplemented with FC2 serum (HyClone FetalClone II from Thermo Scientific, Logan, Utah). In HT-p21-9 cells, p21 expression can be turned on or off using isopropyl-thio-galactosidase (IPTG) [52, 41]. MCF-7 and HCT116 cell lines were obtained from ATCC (Manassas, VA). MCF-7, breast cancer cell line was cultured in high-glucose DMEM (plus pyruvate) with 10% FBS. HCT-Bax/- cells were derived from HCT116, colorectal adenocarcinoma cell line, and were provided by Bert Vogelstein. Rapamycin was obtained from LC Laboratories (MA, USA) and dissolved in DMSO as 5 mM solution. IPTG (Invitrogen) was dissolved in water as 50 mg/ml stock solution and used in cell culture at final concentration of 1.25-50 μg/ml. LY294002, CGK733, metformin, NAC, UO126 were obtained from Sigma-Aldrich.

### Immunoblot analysis

Whole cell lysates were prepared using boiling lysis buffer (1%SDS, 10 mM Tris.HCl, pH 74.). Equal amounts of proteins were separated on 10% or gradient polyacrylamide gels and transferred to nitrocellulose membranes. The following antibodies were used: rabbit anti-phospho-S6 (Ser235/236) and mouse anti-S6, rabbit anti-phospho AKT and anti-Akt from Cell Signaling Biotechnology; anti-actin antibody from Sigma-Aldrich and mouse anti-HIF-1a from BD Biosciences. Secondary anti-rabbit and anti-mouse HRP conjugated antibodies were from Cell Signaling Biotechnology.

### Preparation of conditioned medium

Conditioned medium (CM) from HT-p21-9 cells plated at high density (10^6^ cells per 60 mm dish) and cultured for 4 days. R-CM, cells were cultured in the presence of 500 nM rapamycin. CM+R: 500 nM rapamycin was added to CM after collection of CM.

### Lactate assay

was performed using L-Lactate assay kit from Eton Bioscience Inc (San Diego, CA) according to manufacture's instructions.

### Replicative viability as measure of CS

Cells were plated at high initial density (see Fig. 1A) and cultured for 4-8 days. Then media with floating (dead cells) were removed, cells trypsinized and a small aliquot of attached cells was replated at low cell density in 6 well plates in fresh medium. After 6-8 days, colonies were stained with 1.0 % crystal violet and in replicate wells cells were trypzinised and counted.

## SUPPLEMENTAL FIGURES

Figure S1 Effect of NaOH on CS HT-p21-9 cells were plated at 80,000/well in 96 well plates. On day 1, 0.5 mM or 1 mM NaOH was added. 100 nM rapamycin (Rapa) was used as positive control. Cells were split on day 5 (4 μl out of 200 μl culture per well into 6 well plates) as shown in Fig. 1A. Colonies were grown for 9 days and stained with Crystal Violet.

Figure S2 HT1080 p21-9 cells passaged the day before (fresh culture) or cultured for 2 weeks without change of the medium (old culture) plated at 80000 cells/well in 96-well plates with or without 500 nM rapamycin. On day 4, cells were trypsinized and equal volumes of adherent cells (2%) were re-plated in 6 well plates. After 7 days, colonies were stained with Crystal violet.

Figure S3 HT-p21-9, HCT116 and HCT Bax−/− cells were plated at high density (upper panel) and regular density (lower panel) and on Day 1 was placed in either normoxia (Nor) or hypoxia (Hyp) with or without 100 nM rapamycin (R) or left untreated (C). Cells were lysed on day 1 (D1) and on day 3 (D3). Immunoblot was performed using indicated antibodies.
