# Supplementary figures and images for: Yeast-like chronological senescence in mammalian cells: phenomenon, mechanism and pharmacological suppression
Source: Aging (Albany NY). 2011 Dec 10;3(11):1078–91. doi: 10.18632/aging.100402 (PMC3249453; doi:10.18632/aging.100402)

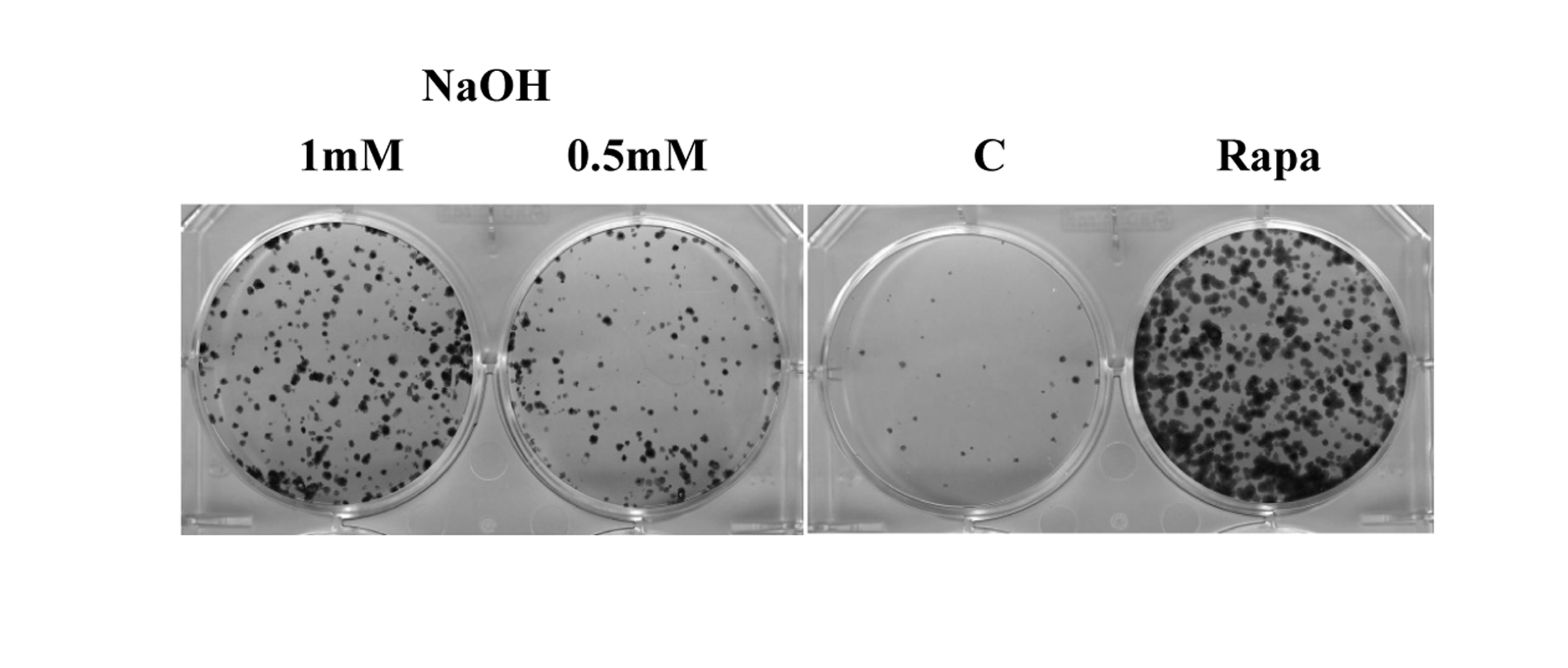

Supplement: Figure S1 — HT-p21-9 cells were plated at 80,000/well in 96 well plates. On day 1, 0.5 mM or 1 mM NaOH was added. 100 nM rapamycin (Rapa) was used as positive control. Cells were split on day 5 (4 μl out of 200 μl culture per well into 6 well plates) as shown in Fig. 1A. Colonies were grown for 9 days and stained with Crystal Violet. [file aging-03-1078-s001.tif]

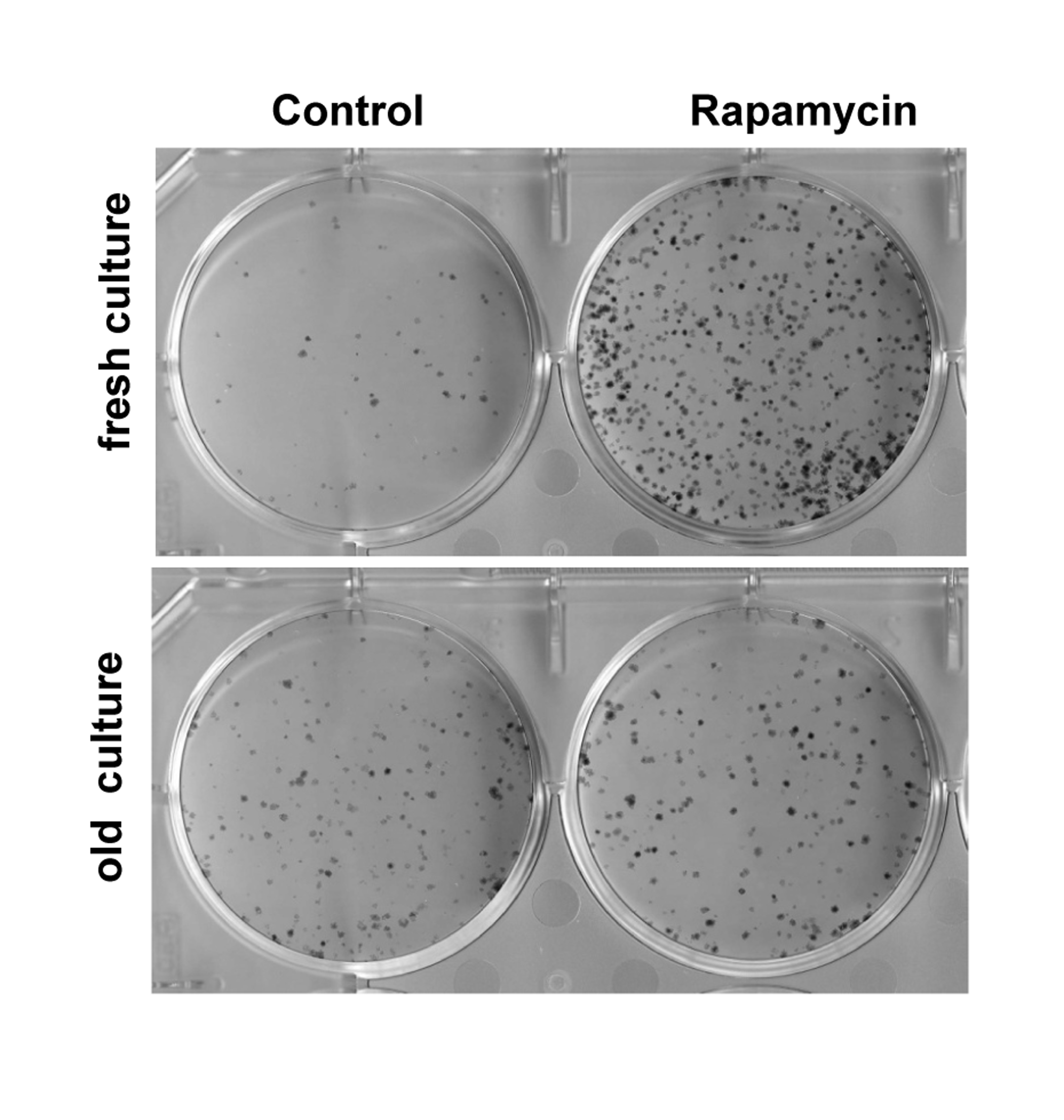

Supplement: Figure S2 — HT1080 p21-9 cells passaged the day before (fresh culture) or cultured for 2 weeks without change of the medium (old culture) plated at 80000 cells/well in 96-well plates with or without 500 nM rapamycin. On day 4, cells were trypsinized and equal volumes of adherent cells (2%) were re-plated in 6 well plates. After 7 days, colonies were stained with Crystal violet. [file aging-03-1078-s002.tif]

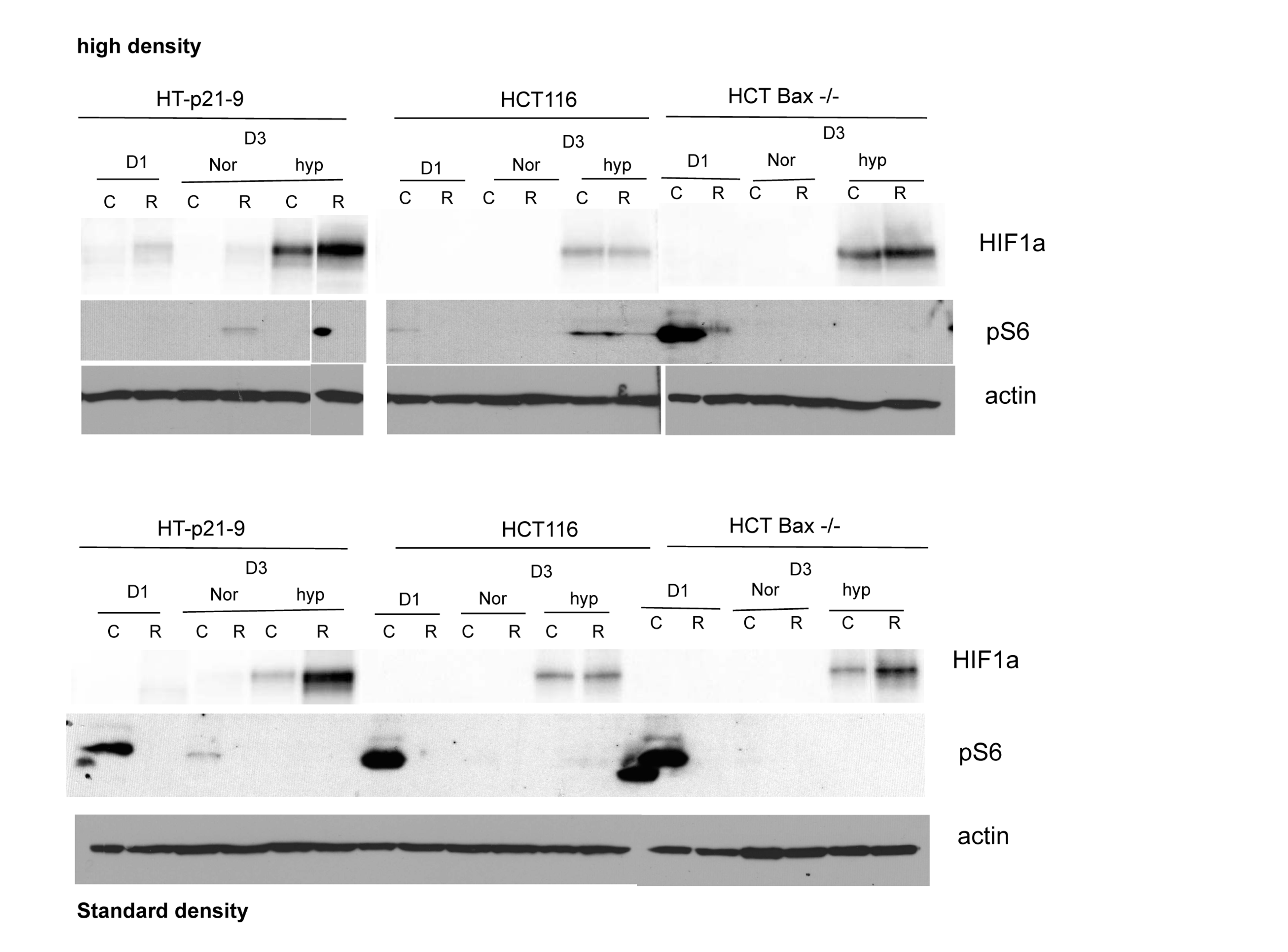

Supplement: Figure S3 — HT-p21-9, HCT116 and HCT Bax−/− cells were plated at high density (upper panel) and regular density (lower panel) and on Day 1 was placed in either normoxia (Nor) or hypoxia (Hyp) with or without 100 nM rapamycin (R) or left untreated (C). Cells were lysed on day 1 (D1) and on day 3 (D3). Immunoblot was performed using indicated antibodies. [file aging-03-1078-s003.tif]
